# A Comprehensive Review of *Punica* *granatum* (Pomegranate) Properties in Toxicological, Pharmacological, Cellular and Molecular Biology Researches

**Published:** 2012

**Authors:** Hamid Reza Rahimi, Mohammad Arastoo, Seyed Nasser Ostad

**Affiliations:** a *Department of Toxicology and Pharmacology, Faculty of Pharmacy and Pharmaceutical Sciences Research Center, Tehran University of Medical Sciences, Tehran, Iran. *; b *Research Center for Science and Technology in Medicine, Tehran University of Medical Sciences, *

**Keywords:** Punica granatum, Chemical components, Toxicological properties, Signaling pathway, Clinical applications

## Abstract

*Punica granatum* (Pg), commonly known as pomegranate (Pg), is a member of the monogeneric family, Punicaceae, and is mainly found in Iran which is considered to be its primary centre of origin. Pg and its chemical components possess various pharmacological and toxicological properties including antioxidant, anti-inflammatory (by inhibiting pro-inflammatory cytokines), anti-cancer and anti-angiogenesis activities. They also show inhibitory effects on invasion/motility, cell cycle, apoptosis, and vital enzymes such as cyclooxygenase (COX), lipooxygenase (LOX), cytochrome P450 (CYP450), phospholipase A2 (PLA2), ornithine decarboxylase (ODC), carbonic anhydrase (CA), 17beta-hydroxysteroid dehydrogenase (17*β*-HSDs) and serine protease (SP). Furthermore, they can stimulate cell differentiation and possess anti-mutagenic effects. Pg can also interfere with several signaling pathways including PI3K/AKT, mTOR, PI3K, Bcl-X, Bax, Bad, MAPK, ERK1/2, P38, JNK, and caspase. However, the exact mechanisms for its pharmacological and toxicological properties remain to be unclear and need further evaluation. These properties strongly suggest a wide range use of Pg for clinical applications. This review will discuss the areas for which Pg has shown therapeutic properties in different mechanisms.

## Introduction

*Punica granatum* (Pomegranate) is a small tree which measures between five and eight meters tall and mainly found in Iran, the Himalayas in northern India, China, USA and throughout the Mediterranean region (1). Pg is one of the important endemic plants of Iran, growing in most regions throughout the country, in arid and semiarid regions due to its ability to adapt to adverse ecological conditions. Over 764 cultivars of *Punica granatum* (Pg) have been collected during a germplasm collection and grown in the cities of Saveh and Yazd (Iran), all of which possess specific fruit characteristics including size, color, taste, time of ripening, and disease resistance (2). The Pg can be also divided into several anatomical compartments including seed, juice, peel, leaf, flower, bark, and root with each possessing interesting pharmacological and toxicological activities. The edible fruit is a berry which is about 5-12 cm in diameter with a rounded hexagonal shape, thick reddish skin and around 600 seeds, each surrounded by a water-laden pulp (aril) ranging in color from white to deep red or purple, the aril is the edible part of the fruit. The seeds are embedded in a white, spongy, astringent pulp (3). According to the holy Quran, pomegranates grow in the gardens of paradise and the Quran has recited the Pg twice as an example of god’s good creations.

The fruit of the Pg has extensively been used as a traditional remedy against acidosis, dysentery, microbial infections, diarrhea, helminth infection, hemorrhage and respiratory pathologies (4). Pg seeds have also been shown to contain the estrogenic compounds, estrone and estradiol (4). Furthermore, the dried pericarp and the juice of the fruit are considered beneficial for treatment of colic, colitis, menorrhagia, oxyuriasis, headache, diuretic, acne, piles, allergic dermatitis, and treatment of oral diseases (5). Recent studies have shown new scientific investigations for the traditional uses of Pg (5). 

*Chemical contents*

Pg contains chemical components in its different compartments, which may possess various pharmacological and toxicological activities (6). These components are summarized in Table 1 (6).

*Antioxidant properties*

Oxidative stress (OS) produces toxic metabolites (7) which can initiate and promote cancers (8, 9). Consumption of polyphenoles and flavonoids are beneficial for the prevention of cardiovascular, inflammatory, and other diseases by preventing OS that induces lipid peroxidation in arterial macrophages and in lipoproteins (10, 11). The presence of antioxidants has been reported in Pg juice (10). Pg contains some species of flavonoids and anthocyanidins (delphinidin, cyaniding and pelargonidin) in its seed oil and juice (6) and shows antioxidant activity three times greater than green tea extract (12). Pg fruit extracts exhibit scavenging activity against hydroxyl radicals (13) and superoxide anions, which could be related to anthocyanidins (6). The antioxidant action of Pg is observed, not only through its scavenging reactions, but also by its ability to form metal chelates (14). Studies have indicated that methanolic extracts from the peel of Pg has a broad spectrum of antioxidant activities which were evaluated by 1,1-diphenyl 2-picrylhydrazyl (DPPH) free radical scavenging, phosphomolybdenum, Ferric (Fe^3+^) Reducing Antioxidant Power (FRAP), and Cupric (Cu^2+^) Reducing Anti-oxidanrt Capacity (CUPRAC) assays (14, 15). Studies have looked at the beneficial effects of pomegranates antioxidant activity *in-vivo* and *in-vitro* and have shown that Pg juice consumption causes a decrease in procarcinogen activation through CYP activity/expression (CYP1A2 and CYP3A) (16), protection of rat gastric mucosa from ethanol or aspirin toxicity (17), protection of neonatal rat brain from hypoxia (18), reduction of hepatic OS (19), reversal of proatherogenic effects which are induced by perturbed shear stress (20), protective effects against UVA- and UVB-induced cell damage and the potential use of Pg polyphenolics in topical applications (21). Other studies have also shown the protective effects of Pg on the cardiovascular system, including reduction of LDL and cholesterol (22, 23), anti-hypertension action by combating OS induced by diabetes and angiotensin II (24), reduction of carotid arterial stenosis and increase of endothelial nitric oxide (NO) syntheses (25, 26); and suggest the Pg as part of a heart-healthy diet through inhibiting of OS mechanism (27).

*Anti-inflammatory effect*

Acute inflammation is a beneficial host response for prevention of tissue injury, but it may also cause immune-associated diseases such as rheumatoid arthritis, inflammatory bowel disease and cancers (28, 29). Interestingly, Pg, Pg has been shown to inhibit inflammation by different mechanisms. 

Cyclooxygenase (COX) and lipooxygenase (LOX), which are key enzymes in the conversion of arachidonic acid to prostaglandins and leukotrienes (important inflammatory mediators), respectively, are inhibited by Pg (30, 31). Non-steroidal anti-inflammatory drugs (NSAIDs) have more adverse effects on cardiovascular function by inhibiting COX and suppressing PGI2 (prostacyclin) in comparison to Pg (32). Ahmed et al. have shown that Pg has a significant inhibitory effect on osteoarthritis (OA) by suppressing the expression of matrix metalloproteinases (MMPs) in OA chondrocyte cultures and preventing collagen degradation. It may also inhibit joint destruction in OA patients (33). Pro-inflammatory cytokines such as IL-1*β* play an important role in OA pathogenesis (34). IL-1*β* induces the expression of MMPs, especially MMP-1 and MMP-13, which are associated to the irreversible breakdown of cartilage matrix through digestion of type-II collagen and the consequent release of matrix proteoglycan from the cartilage (34). Furthermore, Pg has shown anti-inflammatory effects in a colitis rat model (35). However, studies have shown the inhibitory effect of Pg on production of pro-inflammatory cytokines (34-36). These studies demonstrate that Pg inhibits the p38-mitogen-activated protein kinase (p38-MAPK) pathway and transcription factor, NF-kB (nuclear factor kappa-light-chain-enhancer of activated B cells). Activation of p38-MAPK and NF-kB are associated with increased gene expression of TNF-*α*, IL-1*β*, MCP1, iNOS, and COX-2 agents which are critical mediators of inflammation (36). Studies have also shown that administration of 50 mg/kg of Pg extract for 28 days causes a decrease in malondialdehyde (MDA), TNF-*α*, and IL-1*β *levels in rats with liver fibroses (37). Also, Shukla *et al*. showed that pretreatment with 13.6 mg/kg of Pg extract decreased the arthritis incidence and lowered IL-6 and IL-1*β* levels in animal model of rheumatoid arthritis (38).

*Carcinogenesis*

Pg possesses inhibitory effects on different type of cancers such as prostate (39,40), breast (41), colon (42,43), and lung cancers (44). Different mechanisms have been outlined for pomegranates anti-cancer activities in these studies. Pg inhibits NF-kBand cell viability of prostate cancer cell lines in a dose-dependent manner in the LAPC4 xenograft model, *in-vitro* (45). Pg polyphenols, ellagitannin-rich extract and whole juice extract inhibited gene expression of HSD3B2 (3beta-hydroxysteroid dehydrogenase type 2), AKR1C3 (aldo-ketoreductase family 1 member C3) and SRD5A1 (steroid 5alpha reductase type 1), which are key androgen-synthesizing enzymes in LNCaP, LNCaP-AR, and DU-145 human prostate cancer cells (46). Because Pg inhibits CYP activity/expression which is necessary for activation of procarcinogens, it may have anti-carcinogenesis effects (16). Some metabolites of pomegranates chemical components such as 3,8-dihydroxy-6H-dibenzo[b, d]pyran-6-one (urolithin A, UA) which is produced from Ellagitannins (ETs) may also possess anti-cancer effects (46). Treatment with (50-150 μg/mL) pomegranate fruit extract (PFE) for 72 h was found to result in a significant inhibition of lung cancer, with dose-dependent arrest of cells in G_0_/G_1_ phase of the cell cycle, induction of WAF1/p21 and KIP1/p27, decrease in the protein expressions of cyclins D1, D2, and E, decrease in cyclin-dependent kinase (cdk) 2, cdk4 and cdk6 expression, phosphorylation of MAPK proteins, inhibition of PI3K, phosphorylation of Akt at Thr308, NF-kB and IKK (inhibitor of kappa kinase) alpha, degradation and phosphorylation of IκB, Ki-67 and PCNA (44). Also, the levels of Bax and Bcl-2 were altered by PE in PC-3 cell line (47).

*Angiogenesis*

Angiogenesis is an important process for the development of new blood vessels, which is essential in supplying oxygen and nutrition for tumor growth and progression of cancers (48). Therefore, angiogenesis is a possible target for cancer prevention strategy (49, 50). Fibrocytes are important in angiogenesis, as they lay the requisite intracellular infrastructure of blood vessels (51), secrete extracellular matrix degrading enzymes such as MMP-9 which stimulate endothelial cell invasion, and secrete pro-angiogenic factors such as vascular endothelial growth factor (VEGF), basic fibrocyte growth factor (bFGF), and interleukins (IL) (52). Interestingly, recent study has shown the ability of Pg to inhibit angiogenesis (53). Toi*et et al.* evaluated the anti-angiogenic potential of Pg by measuring vascular endothelial growth factor (VEGF), IL-4, and migration inhibitory factor (MIF) in the conditioned media of estrogen sensitive (MCF-7) or estrogen resistant (MDA-MB-231) human breast cancer cells, and immortalized normal human breast epithelial cells (MCF-10A). VEGF was strongly decreased in MCF-10A and MCF-7, however, MIF was increased in MDA-MB-231, showing significant potential for inhibitory effects of angiogenesis by Pg fractions on human umbilical vein endothelial cells (HUVEC) (53).

*Invasion and motility*

Understanding the mechanisms of tumor cell invasion and metastasis could prove to be important for preventing tumor cell spread. MMP-1, MMP-2, and MMP-9, are a family of zinc-dependant endoproteinases which are most closely linked with metastasis of cancer cells (54-57). Activity of MMPs are regulated on certain levels including transcriptional control, proenzyme activation, and inhibition of activated MMPs by non specific inhibitors such as *α*2-macroglobulin (58), and specific endogenous inhibitors such as tissue inhibitors of metalloproteinase (TIMP) (58). TIMPs bind to the active site of MMPs and block access of MMPs to their substrates (58). Caffeic acid phenethyl ester (CAPE), a compound of Pg which is also derived from honey bee propolis has shown dose-dependent decreases in MMP and TIMP-2 mRNA levels in HT1080 human fibrosarcoma cells, as detected by reverse transcriptase-polymerase chain reaction (RT-PCR). Gelatin zymography analysis confirmed this when compared to controls (57). This study shows the role of CAPE as a potent anti-metastatic agent, which can significantly inhibit the metastatic and invasive capacity of malignant cells (57). Pg has shown dose-dependent inhibition effect on NF-kB-dependent reporter gene expression which is associated to proliferation, invasion, and motility in aggressive breast cancer phenotypes. This effect is behind to decrease RhoC and RhoA protein expression, suggests a role for these extracts in lowering the metastatic potential of aggressive breast cancer species (60). Four pure chemicals, ellagic acid, caffeic acid, luteolin, and punicic acid, obtain from the Pg fruit were presented as potential inhibitors of *in-vitro* invasion of human PC-3 prostate cancer cells in an assay employing Matrigel artificial membranes (61). 

*Cell cycle arrest*

The cell cycle is a series of events which takes place in a cell, leading to its division and duplication. It consists of four distinct phases; G1 phase, S phase (synthesis), G2 phase (collectively known as interphase) and M phase (mitosis). Multiple checkpoints have been identified to verify whether the processes at each phase of the cell cycle have been accurately completed before progression into the next phase. Cell cycle may be altered following exposure to Pg. Previous studies have suggested several mechanisms for these effects, such as modulation of cell signaling molecules in the cell cycle machinery. *Punica granatum extrac*t (PE) inhibited the proliferation of mouse mammary cancer cell line (WA4), derived from mouse MMTV-Wnt-1 mammary tumors in a time and concentration-dependent manner through an arrest of cell cycle progression in the G_0_/G_1_ phase (62). Ellagitannins, derived from Pg juice, and their metabolites, urolithins exhibit dose and time-dependent decreases in cell proliferation and clonogenic efficiency of HT-29 cells through cell cycle arrest in the G_0_/G_1_ and G_2_/M stages of the cell cycle followed by induction of apoptosis (43). Moreover, Pg treatment induced a dose-dependent arrest in the G_0_/G_1_ phase of the cell cycle which was assessed by DNA cell cycle analysis in the lung cancer cell line (A549) (63). Pg pretreatment of normal human epidermal keratinocytes (NHEK) has been found to increase the cell cycle arrest induced by UVA in the G_1_ phase of the cell cycle (64). Furthermore, Androgen-independent cell line, DU 145 has shown a significant increase from 11% to 22% in G_2_/M cells (p < 0.05) by treatment with (35 μg/mL) Pg cold-pressed oil (65). Ellagic acid is a phenolic compound, which may belong to Pg, and induces cell cycle arrest and apoptosis in T24 human bladder cancer cells *in-vitro* through induced G_0_/G_1 _phase arrest, increased p53 and p21 and decreased cyclin-dependent kinase (Cdk2) gene expression (65). Cdks as the mainly one Cdk4 is a key key molecule in the regulation of cell cycle progression at the G_1_-S phase restriction point is inhibited by p16 (INK4a), a tumor suppressor. It has been reported that the N-terminal of different truncated p16 (INK4a) molecules is not crucial for the interaction with Cdk4 (66). However, Ostad *et al*. have previously shown that the C-terminal domain of p16 (INK4a) is adequate in inducing cell cycle arrest, growth inhibition, and CDK4/6 interaction (68).

*Apoptosis*

Apoptosis is the process of programmed cell death, which is a useful marker for predicting tumor response after anti-cancer treatment. Pg causes apoptosis by different mechanisms. PE has been found to induce apoptosis by increasing caspase-3 activity in a mouse mammary cancer cell line (WA4) (62). In addition, Pg extracts and punic acid, an omega-5 long chain poly unsaturated fatty acid derived from Pg, have been shown to induce apoptosis in both an estrogen in sensitive breast cancer cell line (MDA-MB-231) and an estrogen sensitive cell line developed from MDA-MB-231 cells (MDA-ERalpha7) through lipid peroxidation and the PKC (Protein kinase C) signaling pathway (69). They also cause disruption to the cellular mitochondrial membrane (69). The relationship between pg-induced apoptosis in human prostate cancer cells (LAPC4) and the IGF/IGFBP system have been investigated (70). POMx (a highly potent Pg extract prepared from skin and arils, minus the seeds) and IGFBP-3 have been shown to synergistically stimulate apoptosis. Inhibition of cell growth resulted in increased JNK phosphorylation and decreased Akt and mTOR activation (70). Pg treatment of normal human epidermal keratinocytes (NHEK) inhibited UVB-mediated activation of MAPK and NF-kB pathways, as well as other signal transducers and activators of the apoptosis pathway including transcription 3 (STAT3), PKB/AKT, ERK1/2, mTOR, PI3K, Bcl-X(L) (antiapoptotic protein), Bax and Bad (proapoptotic proteins) (64). The role of MAPK signaling pathways and effects of PI3K/AKT, ERK1/2, P38, and JNK on epidermal growth factor (EGF) signaling in proliferation of human mesenchymal stem cells (hMSCs) have been shown *in-vitro* (71). The cell growth is controlled by the interaction of survival and cell growth arrest pathways and the activity of survival pathways such as Akt and ERK1/2 with regard to XIAP (inhibitor of apoptosis) in serum starvation has been investigated and the survival role for ERK in serum starvation has been reported (72). Recently, the Pg inhibition of cell growth, followed by apoptosis of highly aggressive human prostate carcinoma PC3 cells through modulations in the cyclin kinase inhibitor-cyclin-dependent kinase machinery have been shown by Malik *et al.* These events were associated to alterations in the levels of Bax and Bcl-2, shifting the Bax: Bcl-2 ratio in favor of apoptosis (47). Ellagitannins (ETs) and hydrolysable tannins, which are found in Pg and their hydrolyzed product, as well as ellagic acid (EA), have been reported to induce apoptosis in human colon cancer Caco-2 cells through down-regulation of cyclins A and B_1_, upregulation of cyclin E, cell-cycle arrest in the S phase, induction of apoptosis via intrinsic pathways (FAS-independent, caspase-8 independent) through bcl-XL down-regulation with mitochondrial release of cytochrome c into the cytosol as well as activation of initiator caspase-9 and effector caspase-3 (73). Induction of Bax and Bak (proapoptotic), down-regulation of Bcl-X(L) and Bcl-2 (anti-apoptotic), induction of WAF1/p21 and KIP1/p27, a decrease in cyclins D1, D2, and E, and a decrease in cdk2, cdk4, and cdk6 expression have been shown to occur in prostate cancer PC3 cells, following Pg treatment (74).

*Punica granatum effects on vital enzymes*

Enzymes are proteins that catalyze biochemical/chemical reactions. Pg has been shown to inhibit different enzymes including phospholipase A2 (PLA2) (that catalytically hydrolyzes the bond releasing arachidonic acid and lysophospholipids) (75), cyclooxygenase (COX), lipooxygenase (LOX) (30), cytochrome P450 (76) and ornithine decarboxylase (ODC) (77) which plays a role in the urea cycle and catalyzes the decarboxylation of ornithine to polyamines such as putrescine. Polyamines regulate growth processes and stimulate the growth of cancer (78). Carbonic anhydrase (CA) that catalyzes the hydration of carbon dioxide to form bicarbonate (HCO_3_^-^) is also inhibited (79). CA inhibitors such as Pg have been shown to inhibit cancer cell growth *in-vitro* and *in-vivo* (80). Aromatase is enzyme responsible for a key step in the biosynthesis of estrogens and catalyzes the formation of estrone and estradiol, which is inhibited by Pg (81). One of the possible mechanisms in which Pg can inhibit breast cancer is its inhibitory effect on aromatase and 17 beta-hydroxysteroid dehydrogenase enzymes (17*β*-HSDs), as well as its anti-estrogenic activity (41). Furthermore, ellagitannins (ET) and urolithin B (UB), which are found in relatively high quantities in Pg, have been shown to most effectively inhibit aromatase activity in a live cell assay (82). Serine protease (SP) is another enzyme which is inhibited by Pg. SP is enzymes in which one of the amino acids in the active site is serine. Protease plays an essential role in modulating the turnover of extracellular matrix (EC), which provides morphological support for cell growth and differentiation (83). Furthermore, protease has a verity of important functions including angiogenesis, vasculogenesis, apoptosis, and cell migration/invasion (84). Ellagic acid and punicalagin, from Pg, have shown lower inhibitory effects on alpha-secretase (TACE) and other serine proteases such as chymotrypsin, trypsin, and elastase, thus indicating that they are relatively specific inhibitors of beta-secretase (BACE1) (85). Other studies have shown that catechin and epicatechin (epigallocatechin-3-gallate) (86-88), which are present in Pg (89), can inhibit SP.

*Cellular differentiation*

Cellular differentiation, in developmental biology, is the process by which a less specialized cell becomes a more specialized cell type. Study has shown that Pg stimulates the differentiation of osteoblastic MC3T3-E1 cells and affects the function of these cells (4). Pg seed oil (but not aqueous extracts of fermented juice, peel or seed cake) has been shown to stimulate keratinocyte proliferation in monolayer culture, without effecting fibroblast function, and as a result facilitates skin repair and promotes regeneration of dermis and epidermis (56). flavonoid-rich fractions from fermented Pg juice and aqueous extraction of Pg pericarps are strong promoters of differentiation in human HL-60 promyelocytic leukemia cells, which are detected by nitro blue tetrazolium reducing activity, non specific esterase activity, specific esterase activity, and phagocytic activity, whereas flavonoid-rich fractions from fresh Pg juice only show a relatively mild differentiation-promoting effect. Furthermore, the effect of Pg on differentiation has been observed in breast and prostate cell lines (90).

*Anti-mutagenicity*

A mutagen is a physical orchemical agent that alters the genetic material of an organism, usually DNA, permanently and thus increases the frequency of mutations above the natural background level Mutagenicity is the capacity of a chemical or physical agent to cause such permanent change. It has been shown that Pg peel fractions, especially methanol, has anti-mutagenic activities as was detected by the Ames Salmonella/microsome assay against sodium azide (NaN3), methyl methane sulphonate (MMS), 2-aminofluorene (2-AF), and benzo(a)pyrene (B(a)P) induced mutagenicity in Salmonella typhimurium (TA97a, TA98, TA100 and TA102) tester strains (15). Methanolic extract of Pg (15 mg/plate) shows the highest anti-mutagenic activity in TA 100 cells (91).

*Clinical application*

Considering the mentioned properties, Pg has the potential to be used in many clinical applications. Studies have shown that Pg inhibits prostate cancer cell growth, induces apoptosis in PC-3 cells (highly aggressive prostate carcinoma cells), suppresses invasion of PC-3 cells and decreases proliferation of DU-145 prostate cancer cells *in-vitro* (52). Treatment of HT-29 colon cancer cells has been indicated by Pg juice through decreasing COX-2 expression and inhibiting inflammatory cell signaling processes which may cause cancer initiation and progression (92). Furthermore, COX-2 is involved in the proliferative response of human periodontal fibroblast (HPLF) cells to Emdogain (EMD) (93). There has been a correlation between inducible nitric oxide (iNOS) synthase and COX-2 expression in human colorectal adenocarcinoma. In fact, a possible link between advanced stages of this disease and higher expression of iNOS and COX-2 has been shown by Habibollahi *et al.* (94).

**Table 1:** Phytochemicals present in *Punina granatum* (pomegranate)

**Pomegranate Phytochemicals**	**Formula**	**Molecular weight (MW)**	**Plant Part**
***Ellagitannins and Gallotannins***			
2,3-(S)-HHDP-D-glucose^a^	C_20_H_18_O_14_	482.35	Bark, peel
Castalagin	C_41_H_26_O_26_	934.63	Bark
Casuariin	C_34_H_24_O_22_	784.54	Bark
Casuarinin		936.65	Bark, pericarp
Corilagin	C_27_H_22_O_18_	634.45	Fruit, leaves, pericarp
Cyclic 2,4:3,6-bis(4,4′,5,5′,6,6′-hexahydroxy [1,1′-biphenyl]- 2,2′-dicarboxylate) 1-(3,4,5-trihydroxybenzoate) b-D-Glucose	C_41_H_28_O_26_	936.65	leaves
Granatin A	C_34_H_24_O_23_	800.54	Pericarp
Granatin B	C_34_H_28_O_27_	952.64	Peel
Pedunculagin	C_34_H_24_O_22_	784.52	Bark, pericarp
Punicacortein A	C_27_H_22_O_18_	634.45	Bark
Punicacortein B	C_27_H_22_O_18_	634.45	Bark
Punicafolin	C_41_H_30_O_26_	938.66	Leaves
Punigluconin	C_34_H_26_O_23_	802.56	Bark
Strictinin	C_27_H_22_O_18_	634.45	Leaves
Tellimagrandin I	C_34_H_26_O_22_	786.56	Leaves, pericarp
Tercatain	C_34_H_26_O_22_	786.56	Leaves
2-O-galloyl-4,6(S,S) gallagoyl-D-glucose	C_41_H_26_O_26_	934.63	Bark
5-O-galloyl-punicacortein D	C_54_H_34_O_34_	1222.8	Leaves
Punicacortein C	C_47_H_26_O_30_	1070.7	Bark
Punicacortein D	C_47_H_26_O_30_	1070.7	Bark, heartwood
Punicalin	C_34_H_22_O_22_	782.53	Bark, pericarp
Punicalagin	C_48_H_28_O_30_	1084.7	Bark, pericarp, peel
Terminalin/gallayldilacton	C_28_H_20_O_16_	602.37	Pericarp
***Ellagic Acid Derivatives***			
Ellagic acid	C_14_H_6_O_8_	302.19	Fruit, pericarp, bark
Ellagic acid, 3,3′-di-O-methyl	C_16_H_10_O_8_	330.25	Seed
Ellagic acid, 3,3′, 4′-tri-O-methyl	C_17_H_12_O_8_	344.27	Seed
Ellagic acid, 3′-O-methyl-3, 4-methylene	C_16_H_8_O_8_	328.23	Heartwood
Eschweilenol C	C_20_H_16_O_12_	448.33	Heartwood
Diellagic acid rhamnosyl(1-4) glucoside	C_40_H_30_O_24_	894.65	Heartwood
***Catechin and Procyanidins***			
(-)-Catechin	C_15_H_14_O_6_	290.27	Juice
Catechin-(4,8)-gallocatechin	C_30_H_26_O_13_	594.52	Peel
Gallocatechin	C_15_H_14_O_7_	306.27	Peel
Gallocatechin-(4,8)-catechin	C_30_H_26_O_13_	594.52	Peel
Gallocatechin-(4,8)-gallocatechin	C_30_H_26_O_14_	610.52	Peel
Procyanidin B1	C_30_H_26_O_12_	578.52	Juice
Procyanidin B2	C_30_H_26_O_12_	578.52	Juice
***Anthocyanins and Anthocyanidins***			
Cyanidin	C_15_H_11_O_6_	287.24	Peel
Cyanidin-3-glucoside	C_21_H_21_O_11_	449.38	Juice
Cyanidin-3,5-diglucoside	C_27_H_31_O_16_	611.52	Juice
(Continued)			
Cyanidin-3-rutinoside	C_27_H_31_O_15_	595.53	Juice
Delphinidin	C_15_H_11_O_7_	303.24	Juice
Delphinidin-3-glucoside	C_2_1H_21_O_12_	465.38	Juice
Delphinidin 3, 5-diglucoside	C_27_H_31_O_17_	627.52	Juice
Pelargonidin 3-glucoside	C_21_H_21_O_10_	433.38	Juice
Pelargonidin 3,5-diglucoside	C_27_H_31_O_15_	595.53	Juice
Flavonols			
Apigenin-4′-O-*β*-D-glucoside	C_21_H_20_O_11_	448.32	Leaves
Kaempferol	C_15_H_10_O_6_	286.24	Peel, fruit
Luteolin	C_15_H_10_O_6_	286.24	Peel, fruit
Luteolin-3′-O-*β*-D-glucoside	C_21_H_20_O_10_	432.11	Leaves
Luteolin-4′-O-*β*-D-glucoside	C_21_H_20_O_10_	432.11	Leaves
Luteolin-3′-O-*β*-D-Xyloside	C_21_H_18_O_10_	418.09	Leaves
Myricetin	C_15_H_10_O_8_	318.04	Fruit
Quercetin	C_15_H_10_O_7_	302.04	Peel, fruit
Quercimeritrin	C_21_H_20_O_12_	464.38	Fruit
Quercetin-3-O-rutinoside	C_27_H_30_O_16_	610.52	Fruit
Quercetin-3,4′-dimethyl ether 7-O-*α*-L-arabinofuranosyl-(1-6)-*β*-D-glucoside	C_28_H_32_O_16_	624.54	Bark, peel
Eriodictyol-7-O-*α*-Larabinofuranosyl (1-6)-*β*-D-glucoside	C_26_H_30_O_15_	582.51	Leaves
Naringenin 4′-methylether 7-O-*α*-L-arabinofuranosyl (1-6)-*β*-D-glucoside	C_27_H_32_O_14_	580.53	Leaves
Organic Acids			
Caffeic acid	C_9_H_8_O_4_	180.16	Juice
Chlorogenic acid	C_16_H_18_O_9_	345.31	Juice
Cinnamic acid	C_9_H_8_O_2_	148.16	Juice
Citric acid	C_6_H_8_O_7_	192.12	Juice
o-Coumaric acid	C_9_H_8_O_3_	164.16	Juice
p-Coumaric acid	C_9_H_8_O_3_	164.16	Juice
Ferulic acid	C_10_H_10_O_4_	194.18	Juice
Gallic acid	C_7_H_6_O_5_	170.12	Juice
L-Malic acid	C_4_H_6_O_5_	134.09	Juice
Oxalic acid	C_2_H_2_O_4_	90.03	Juice
Protocatechuic acid	C_7_H_6_O_4_	154.12	Juice
Quinic acid	C_7_H_12_O_6_	192.17	Juice
Succinic acid	C_4_H_6_O_4_	118.09	Juice
Organic Acids			
Caffeic acid	C_9_H8O_4_	180.16	Juice
Chlorogenic acid	C_16_H_18_O_9_	345.31	Juice
Cinnamic acid	C_9_H_8_O_2_	148.16	Juice
Citric acid	C_6_H_8_O_7_	192.12	Juice
o-Coumaric acid	C_9_H_8_O_3_	164.16	Juice
p-Coumaric acid	C_9_H_8_O_3_	164.16	Juice
Ferulic acid	C_10_H_10_O_4_	194.18	Juice
Gallic acid	C_7_H_6_O_5_	170.12	Juice
L-Malic acid	C_4_H_6_O_5_	134.09	Juice
Oxalic acid	C_2_H_2_O_4_	90.03	Juice
Protocatechuic acid	C_7_H_6_O_4_	154.12	Juice
(Continued)			
Quinic acid	C_7_H_12_O_6_	192.17	Juice
Succinic acid	C_4_H_6_O_4_	118.09	Juice
Tartaric acid	C_4_H_6_O_6_	150.09	Juice
Simple Gallyol Derivatives			
Brevifolin	C_12_H_8_O_6_	248.19	Leaves
Brevifolin carboxylic acid	C_13_H_8_O_8_	292.2	Leaves
Brevifolin carboxylic acid-10-monosulphate	C_13_H_7_KO_10_S	394.25	Leaves
1,2,3-Tri-O-galloyl-*β*-D-glucose	C_27_H_24_O_18_	448.32	Leaves
1,2,4-Tri-O-galloyl-*β*-D-glucose	C_27_H24O18	286.24	Leaves
1,2,6-Tri-O-galloyl-*β*-D-glucose	C_27_H_24_O_18_	286.24	Leaves
1,4,6-Tri-O-galloyl-*β*-D-glucose	C_27_H_24_O_18_	432.11	Leaves
1,3,4-Tri-O-galloyl-*β*-D-glucose	C_27_H_24_O_18_	432.11	Leaves
1,2, 4, 6-Tetra-O-galloyl-*β*-D-glucose	C_34_H_28_O_22_	418.09	Leaves
1,2,3,4, 6-Pent-O-galloyl-*β*-D-glucose	C_41_H_32_O_26_	318.04	Leaves
Methyl gallate	C_8_H_8_O_5_	302.04	Heratwood
3,4,8,9,10-pentahydroxy-dibenzo[b,d]pyran-6-one	C_13_H_8_O_7_	464.38	Leaves
Fatty Acids and Triglycerides			
Eicosenoic acid	C_20_H_40_O_2_	312.53	Seed oil
Linoleic acid	C_18_H_32_O_2_	280.45	Seed oil
Linolenic acid	C_18_H_30_O2	278.43	Seed oil
Oleic acid	C_18_H_34_O2	282.46	Seed oil
Palmitic acid	C_16_H_32_O_2_	256.42	Seed oil
Punicic acid	C_18_H_30_O_2_	278.43	Seed oil
Stearic acid	C_18_H_36_O_2_	284.48	Seed oil
Tri-O-punicylglycerol	C_57_H_92_O_6_	873.34	Seeds
Di-O-punicyl-O-octadeca-8Z-11Z-13E-trienylglycerol	C_57_H_92_O_6_	873.34	Seeds
1-O-trans, cis, trans, octadecatrienol glycerol	C_21_H_36_O_4_	352.51	Seed oil
1-O-isopentyl-3-O-octadec-2-enoyl glycerol	C_26_H_50_O_4_	426.67	Seed oil
Sterols and Terpenoids			
Asiatic acid	C_30_H4_8_O_5_	488.7	Juice
Betulinic acid	C_30_H_48_O_3_	456.70	Seed
Cholesterol	C_27_H_46_O	386.65	Seed oil
Daucosterol	C_35_H_60_O_6_	576.85	Seed
Estrone	C_18_H_22_O_2_	270.37	Seed oil
Estradiol	C_18_H_24_O_2_	272.38	Seed oil
Estriol	C_18_H_24_O_3_	288.38	Seed oil
Friedooleanan-3-one	C_30_H_50_O	426.72	Bark
*β*-Sitosterol	C_29_H_50_O	414.71	Seed oil, leaves, stem
Stigmasterol	C_29_H_48_O	412.69	Seed oil
Testosterone	C_19_H_28_O_2_	288.42	Seed oil
Ursolic acid	C_30_H_48_O_3_	456.70	Seed
Alkaloids			
Hygrine	C_8_H_15_NO	141.21	Root bark
(Continued)			
Norhygrine	C_7_H_13_NO	127.18	Root bark
Pelletierine	C_8_H_15_NO	141.21	Bark
N-methyl pelletierine	C_9_H_17_NO	155.24	Bark
Sedridine	C8H17NO	143.23	Bark
Pseudopelletierine	C_9_H_15_NO	153.22	Bark
Nor-pseudopelletierine	C_8_H_13_NO	139.19	Bark
2,3,4,5-tetrahydro-6-propenyl-pyridine	C_8_H_13_N	123.20	Bark
3,4,5,6-tetrahydro-a-methyl-2-pyridine ethanol	C_8_H_15_NO	141.21	Bark
1-(2,5-dyihydroxy-phenyl)-pyridium chloride	C_11_H_10_ClNO_2_	223.66	Leaves
Other Compounds			
Coniferyl 9-O-[*β*-D-apiofuranosyl-(1-6)]-O-*β*-D-glucopyranoside	C_21_H_30_O_12_	474.46	Seed
Pseudopelletierine	C_9_H_15_NO	153.22	Bark
Nor-pseudopelletierine	C_8_H_13_NO	139.19	Bark
2,3,4,5-tetrahydro-6-propenyl-pyridine	C_8_H13N	123.20	Bark
3,4,5,6-tetrahydro-a-methyl-2-pyridine ethanol	C_8_H_15_NO	141.21	Bark
1-(2,5-dyihydroxy-phenyl)-pyridium chloride	C_11_H_10_ClNO_2_	223.66	Leaves
Other Compounds			
Coniferyl 9-O-[*β*-D-apiofuranosyl-(1-6)]-O-*β*-D-glucopyranoside	C_21_H_30_O_12_	474.46	Seed
Sinapyl 9-O-[*β*-D-apiofuranosyl-(1-6)]-O-*β-*D-glucopyranoside	C_18_H_36_O_2_	284.48	Seed
Phenylethylrutinoside	C_57_H_92_O_6_	873.34	Seeds
Icariside D1	C_57_H_92_O_6_	873.34	Seeds
Mannitol	C_21_H_36_O_4_	352.51	Bark

a HHDP =hexahydroxydiphenoyl

Pg has been shown to inhibit breast cancer cell lines MCF-7 and MB-MDA-231 by hindering angiogenesis, tumor growth, invasiveness, proliferation, and induction of apoptosis (53, 95-97). An inhibitory effect of Pg has been shown on lung and skin cancer models (44, 98). Moreover, Wongwattanasathien *et al.* have shown the inhibitory effect of Pg on HL-60 human leukemia cells through inhibition of proliferation and differentiation of these cell lines (91). Pg has been shown to have anti-atherosclerotic effects through protection of endothelial function, destructive effects on reactive oxygen species such as NO by its antioxidant properties (99), increase MPM uptake of oxidized LDL, and decrease of lipid peroxidation and cholesterol levels (100). Furthermore, Pg has been shown to have anti-hyperlipidemia activity through activation of peroxisome proliferators activated receptor (PPAR-*α*), which can decrease cardiac uptake and circulating lipids (101). Furthermore, Pg decreases cholesterol levels by decreasing absorption and increasing fecal excretion of cholesterol, as well as effecting cholesterol metabolism through HMG-CoA reductase and sterol O-acyltransfrase (102). It has also been demonstrated that Pg has anti-hypertensive effects, with inhibited serum angiotensin converting enzyme (ACE) and decreased systolic blood pressure being observed in hypertensive patients (103). Reduced myocardial ischemia and improved myocardial perfusion were also caused by Pg (104). In an animal model of diabetes, Pg resulted in lower serum C-peptides, a pro-insulin metabolite marker for endogenously secreted insulin, by 23 percent compared to baseline levels in diabetic patients (105). Pg has also shown effectiveness in controlling oral inflammation (106) as well as bacterial (107) and fungal counts in periodontal diseases (108), and Candida associated denture stomatitis (109). Pg has been found to have antibacterial properties against oral bacteria (106, 109), including methicilin-resistance *Staphylococcus aureus* (MRSA) and methicillin-sensitive *Staphylococcus aureus* (MSSA) (110). Kasai *et al*. have also shown the protective properties of Pg against UV-induced damage and skin pigmentation, compared to the placebo (111). Azadzoi *et al.* found anti-erectile dysfunction properties for Pg, measured by intracavernous blood flow and penile erection in a rabbit model of arteriogenic erectile dysfunction (112).Therefore, it could be used in male infertility to improve epidermal sperm concentration, sperm motility, decrease the number of abnormal sperms, and increase spermatogenic cell density (113). Pg hasneuroprotective properties in neonatal hypoxia-ischemic brain injury (18, 114) and preventive effects on Alzheimer’s disease, as shown in animal models (improved learning of water maze tasks) (115). Anti-obesity effects (116) have also been described for Pg. COX-2 has been involved in spatial memory retention and may impair the memory by injecting it intra-hippocampal, in rats (117). The inhibitory effect of Pg on COX-2 could be valuable in inhibiting memory impairment.

## Conclusions

In this review, we gathered all the published studies on Pg without date elimination. However attempts were made to explain the new data. Iran is considered to be the primary origin of Pg. Pg juice, fruit, and extracts have been used extensively in the folk medicine of ancient cultures for various medicinal properties (118). Pg has been shown to possess phytochemicals which may hold pharmacological and toxicological properties (6). Nevertheless, the exact effects and involved mechanisms for the pharmacological and toxicological effects of many of these chemicals remain to be cleared. Nowadays, the use of herbal products or medicinal plants, because of their safety and efficiency in the prevention and/or treatment of several chronic diseases, are being extensively investigated worldwide. 

The information presented in this review article which was obtained from *in-vitro*, *in-vivo* and clinical trial investigations has shown some of the pharmacological and toxicological mechanisms and properties of PE. These properties include anti-oxidative, anti-inflammatory, anticancer, anti-angiogenesis, and inhibitory effect on invasion/motility, cell cycle arrest, apoptosis, stimulation of cell differentiation and anti-mutagenic effects, and inhibitory effects on vital enzymes such as COX, LOX, CYP450, PLA_2_, ODC, CA, 17*β*-HSDs and SP. Existence of these pharmacological and toxicological mechanisms and properties and interference of several signaling pathways including PI3K/AKT, mTOR, PI3K, Bcl-X, Bax, Bad, MAPK, ERK1/2, P38, JNK, and caspase relation to Pg, suggest that Pg can be extensively used as a possible therapy for prevention and treatment of several types of diseases including prostate cancer, colon cancer, breast cancer, lung cancer, skin cancer, leukemia, anti-atherosclerosis, hyperlipidemia, hypertension, myocardial ischemia, myocardial perfusion, diabetes, oral inflammation, infection, anti-erectile dysfunction, male infertility, neonatal hypoxia-ischemic brain injury, alzheimer and obesity.
